# The association between the parameters of uroflowmetry and lower urinary tract symptoms in prostate cancer patients after robot-assisted radical prostatectomy

**DOI:** 10.1371/journal.pone.0275069

**Published:** 2022-10-06

**Authors:** Yuta Takeshima, Yuta Yamada, Kotaro Takemura, Naoki Kimura, Yuji Hakozaki, Jimpei Miyakawa, Satoru Taguchi, Yoshiyuki Akiyama, Yusuke Sato, Taketo Kawai, Daisuke Yamada, Tetsuya Fujimura, Haruki Kume

**Affiliations:** 1 Division of Innovative Cancer Therapy, Advanced Research Center, The Institute of Medical Science, The University of Tokyo, Tokyo, Japan; 2 Department of Urology, Graduate School of Medicine, The University of Tokyo, Tokyo, Japan; 3 Department of Urology, Jichi Medical University, Tochigi, Japan; University Medical Center of Princeton at Plainsboro, UNITED STATES

## Abstract

**Objective:**

To investigate changes in uroflowmetry parameters in men undergoing robot-assisted radical prostatectomy (RARP) for prostate cancer.

**Materials and methods:**

Four hundred and twenty-eight patients received uroflowmetry testing before and after RARP from November 2011 to December 2018. Clinicopathological data, including age, initial prostate-specific antigen (PSA), prostate volume, clinical stage, body mass index (BMI), uroflowmetry parameters, and core lower urinary tract symptom scores (CLSS) were retrospectively obtained from clinical records. Changes in uroflowmetry parameters were analyzed for statistical predictors and effects on post-operative outcomes.

**Results:**

A significant increase in maximum flow rate (MFR) and decreases in voided volume (VV) and post-void residual urine (PVR) were seen. In multivariate analysis, age was a negative predictor of MFR increase, while prostate volume was a positive predictor of PVR decrease and MFR increase. VV decrease led to worse incontinence symptoms, while PVR decrease and MFR increase led to improvement in voiding symptoms such as slow stream and straining. Continence recovery curves showed that VV decrease were associated with a delay in continence recovery.

**Conclusions:**

Significant changes were seen in uroflowmetry results after RARP, each parameter directly related to urinary symptoms. In particular, VV decrease was associated with a worsening of incontinence symptoms and continence recovery.

## Introduction

Prostate cancer is the most common malignancy and the second leading cause of cancer death in men in the United States [1]. Robot-assisted radical prostatectomy (RARP) has become the most prevalent treatment modality for localized prostate cancer in the past two decades [2]. Owing to multiple studies conducting pre-and post-RARP urodynamic studies, we have gained knowledge about the influence surgery has on lower urinary tract symptoms (LUTS) and the underlying mechanism [3–7]. However, urodynamic analysis is an invasive and time-consuming test, and it has been pointed out that it should not be conducted routinely in the absence of value in a clinical setting [8]. Uroflowmetry (UFM), on the other hand, is a non-invasive procedure which measures natural micturition parameters and can be easily conducted on a routine basis with minimal burden on the patient. Unfortunately, previous studies of UFM conducted before and after prostatectomy have concentrated on the parameter changes, and very few have established a connection to clinical results such as post-operative incontinence or quality of life (QOL). In this study, we examined these changes in UFM parameters before and after RARP, and further explored their clinical significance, firstly by examining the pre-operative factors that affected these changes, and secondly by examining how these changes in turn affected clinical outcomes such as incontinence recovery and questionnaire results.

## Materials and methods

### Patient population

**Fig 1:**
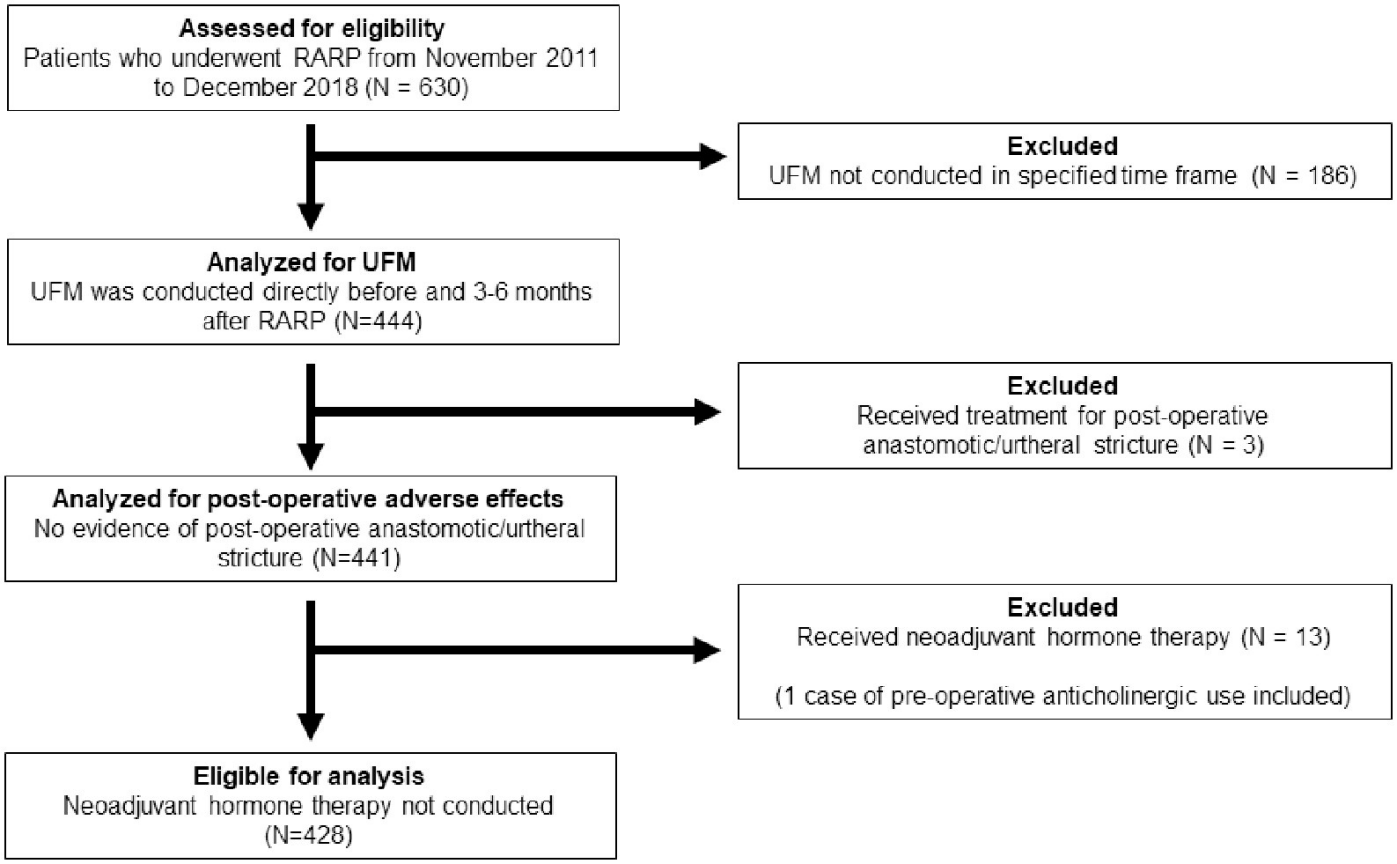
Diagram of patient selection.

A total of 630 patients underwent RARP at the University of Tokyo Hospital from November 2011 to December 2018. RARP was performed by multiple experienced surgeons using da Vinci^®^Si or Xi Surgical Systems (Intuitive Surgical, Sunnydale, CA, USA) by a transperitoneal approach as described in our previous studies [9]. Patients with non-metastatic prostate cancer with or without neoadjuvant hormonal therapy were treated with RARP. Of these patients, UFM was conducted immediately before and 3 to 6 months after RARP in 444 cases. Three patients who developed post-operative anastomotic/urtheral stricture and received treatment were excluded due to effects on micturition parameters from treatment of stricture. Thirteen patients who received neoadjuvant hormone therapy were excluded due to possible effects on baseline parameters. Overall, a total of 428 patients were eligible for the final analysis (Fig 1). In addition to the UFM data, clinicopathological data for baseline parameters such as age, body mass index (BMI), initial prostate-specific antigen (PSA), prostate volume on ultrasound (PV), PSA density (PSAD), D’Amico risk classification, comorbidities such as hypertension and diabetes mellitus, intra-operative parameters such as console time, blood loss, and nerve-sparing, and post-operative data such as pathological T stage, International Society of Urological Pathology (ISUP) grades, and continence status were obtained from our clinical records, prospectively collected in a customized database, and retrospectively analyzed. In all patients, we evaluated the core lower urinary tract symptom score (CLSS), a questionnaire validated for the assessment of LUTS [10], before and after RARP. The CLSS scores obtained 3 to 6 months after RARP were extracted for this study. The clinical and pathological stages of prostate cancer were determined using the American Joint Committee on Cancer TNM staging system (8^th^ edition). Routine follow-ups were conducted at 2 weeks, 1, 3, 6, 12 months post-discharge, and on a cycle of 6 to 12 months thereafter. All patients provided a written informed consent. This study was in accordance with the declaration of Helsinki and was approved by the institutional review board of the Tokyo University Hospital (approval no. 3124).

### Statistical analyses

Wilcoxon rank-sum tests were used to compare continuous values between two groups. Categorical values were analyzed by Pearson’s chi-square test (χ^2^) and Fisher’s exact test. Waterfall plots were generated for the changes in each UFM parameter. Univariate and multivariate analyses using logistic regression models were performed to evaluate which clinical parameters were associated with peri-operative changes in UFM results. Cutoff values used for logistic regression were determined from parameter interquartiles and rounded to the nearest clinically significant value. The cohort was then stratified by the risk factors identified by multivariate analysis, and arranged in box-plots. Cutoffs for box-plots were determined by the Youden index obtained from the receiver operating characteristic (ROC) curve and rounded to the nearest clinically significant value. The association between peri-operative changes in UFM parameters and CLSS questionnaire results were analyzed by Wilcoxon nonparametric test. Kaplan-Meier cumulative event curves were drawn for post-operative continence recovery, and the log-rank test was performed to compare recovery between groups. The cutoff value for these groups were determined from parameter interquartiles and rounded to the nearest clinically significant value, similarly to the logistic regression analysis. A P-value of < 0.05 was considered statistically significant. All statistical analyses were performed using JMP Pro^®^ software, version 14.2 (SAS, Cary, NC, USA).

## Results

**Fig 2:**
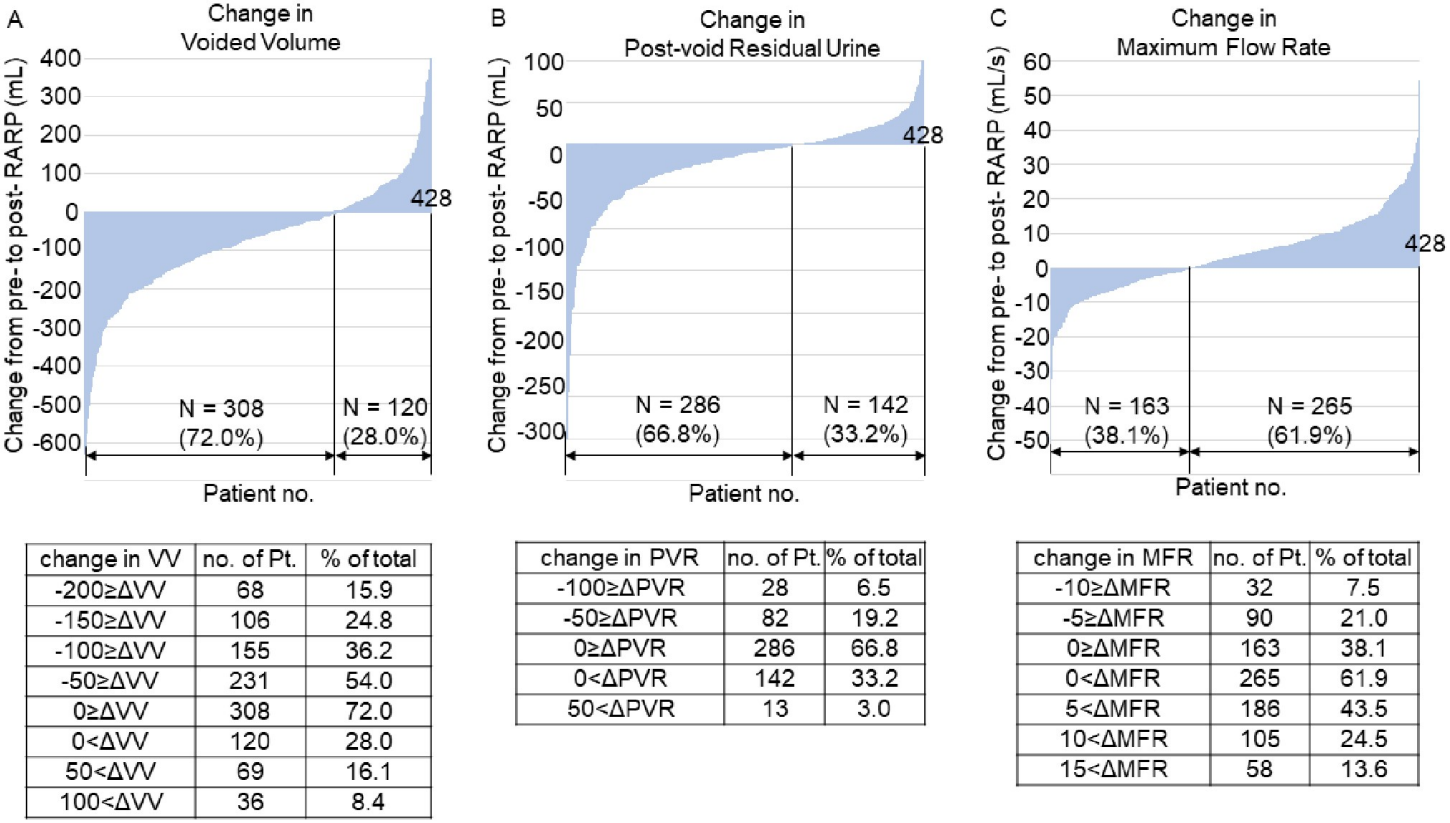
Changes between pre- and post-RARP uroflowmetry parameters. Waterfall plots of changes in (A) voided volume, (B) post-void residual urine, and (C) maximum flow rate. The tables below detail the exact number of patients and percentage of total in groups stratified by values of clinical significance. Abbreviations RARP: robot-assisted radical prostatectomy, ΔVV: perioperative change in voided volume, ΔPVR: perioperative change in post-void residual urine, ΔMFR: perioperative change in maximum flow rate.

Demographics of the subjects and results of UFM pre- and post- RARP are shown in Table 1A. Comparison of pre- and post-RARP UFM parameters showed a significant increase in maximum flow rate (MFR), and significant decreases in voided volume (VV) and post-void residual urine (PVR). Median MFR increased from 14.4 to 17.1 mL/s, median VV decreased from 219 to 144 mL, and median PVR decreased from 37 to 26 mL (P values < 0.001 for all three parameters). These tendencies were confirmed in waterfall plots of the change in each UFM parameter (Fig 2). VV decreased by over 150 mL in 106 patients (24.8%). PVR decreased by over 50 mL in 82 patients (19.2%). MFR increased by over 10 mL/s in 106 patients (24.8%). Using these outcomes, we further analyzed the baseline demographics for each group as stratified by the outcomes of these perioperative changes in each UFM parameter (S1–S3 Tables).

**Table 1:** Patient demographics.

Parameters (N = 428)	Baseline and Pre-RARP	Post-RARP	P value
Age (years)	68(63–71)		
Pre-operative PSA (ng/mL)	7.5(5.6–10.9)		
Prostate volume (mL)	27(21–37)		
PSA density (ng/mL^2^)	0.28(0.19–0.40)		
BMI (kg/m^2^)	23.7(22.0–25.3)		
D’Amico risk classification			
Low	64(15.0%)		
Intermediate-high	364(85.0%)		
Pre-operative α1 blocker			
No	394(92.1%)		
Yes	34(7.9%)		
HT			
absent	248(57.9%)		
present	180(42.1%)		
DM			
absent	361(84.3%)		
present	67(15.7%)		
Console time (min)	169(130–204)		
Blood loss (mL)	250(100–500)		
Nerve sparing			
none	301(70.3%)		
unilateral	122(28.5%)		
bilateral	5(1.2%)		
pT stage			
T2		297(69.4%)	
T3		131(30.6%)	
UFM parameters (mL)			
MFR	14.4(9.9–19.7)	17.1(11.8–24.4)	**< 0.001** *
VV	219 (143–328)	144(89–228)	**< 0.001** *
PVR	37(21–67)	26(15–43)	**< 0.001** *
CLSS			
total	6(3–8)	8 (5–10)	**< 0.001** *
QOL index	3(2–4)	3(2–5)	**< 0.001** *

*: statistically significant

Median value(IQR) or number of cases(%)

Abbreviations RARP: robot-assisted radical prostatectomy, PSA: prostate-specific antigen,

BMI: body mass index, HT: hypertension, DM: diabetes mellitus, pT stage: pathological T stage,

UFM: uroflowmetry, MFR: maximum flow rate, VV: voided volume, PVR: post-void residual urine,

CLSS: core lower urinary tract symptom score, QOL index: quality of life index

CLSS total score and QOL index were significantly increased post-RARP, representing a worsening of LUTS (Table 1, P value < 0.01). As for the individual parameters, CLSS results showed a significant worsening of daytime and nighttime frequency, urgency and stress incontinence, straining, and urethral pain. Conversely, there was a significant improvement in the slow stream parameter (Table 2, P value < 0.01).

**Table 2:** Comparison of pre- and post- operative results of CLSS questionnaire.

	pre-RARP	post-RARP	P value
Daytime frequency	0.96	1.16	**< 0.01** *
Nocturia	1.19	1.55	**< 0.01** *
Urgency	0.74	0.81	0.201
Urgency incontinence	0.17	0.70	**< 0.01** *
Stress incontinence	0.04	1.29	**< 0.01** *
Slow stream	1.19	0.86	**< 0.01** *
Straining	0.57	0.87	**< 0.01** *
Incomplete emptying	0.74	0.67	0.245
Bladder pain	0.10	0.12	0.332
Urethral pain	0.10	0.17	**0.018** *
Total CLSS Score	5.79	8.10	**< 0.01** *

*: statistically significant

Values represent mean CLSS parameter score (larger numbers = stronger symptoms)

**Fig 3:**
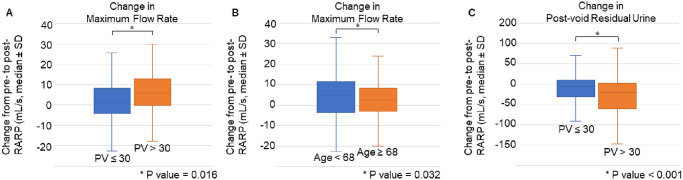
Changes in uroflowmetry results stratified by predictive factors. Box-plots of (A) perioperative change in maximum flow rate when stratified by prostate volume, (B) perioperative change in maximum flow rate when stratified by age, and (C) perioperative change in post-void residual volume when stratified by prostate volume. Median, interquartile, and standard deviation are shown. A significant difference was seen in each parameter when stratified by their respective predictive factors. Abbreviations RARP: robot-assisted radical prostatectomy, PV: prostate volume.

Predictors of these changes in UFM parameters were analyzed by logistic regression as shown in Table 3. For a decrease in VV of greater than 150 mL, none of the examined parameters exhibited a significant relation. For a decrease in PVR of greater than 50 mL, univariate analysis identified PV, console time, and blood loss as positive predictors. In multivariate analysis, PV remained the sole significant predictor of PVR decrease. For an increase in MFR of greater than 10 mL/s, univariate analysis identified age, PV, and BMI as significant predictors. In multivariate analysis, age and PV remained significant predictors of MFR increase. When divided into groups using these predictors, a statistically significant difference in PVR or MFR between the groups was seen in each (Fig 3).

**Table 3:** Analysis of risk factors for perioperative change in uroflowmetry parameters.

	ΔVV (<-150mL vs. ≥ -150mL)						ΔPVR (<-50mL vs. ≥ -50mL)						ΔMFR (> +10mL/s vs. ≤ +10mL/s)					
	Univariate			Multivariate			Univariate			Multivariate			Univariate			Multivariate		
	OR	95%CI	P value	OR	95%CI	P value	OR	95%CI	P value	OR	95%CI	P value	OR	95%CI	P value	OR	95%CI	P value
Age	0.973	0.939–1.009	0.133	0.976	0.942–1.024	0.189	1.027	0.986–1.072	0.199				0.962	0.929–0.997	**0.035** *	0.954	0.919–0.990	**0.013** *
Pre-op PSA	0.979	0.938–1.012	0.239				1.023	0.992–1.053	0.140	1.017	0.986–1.048	0.295	0.991	0.955–1.022	0.603			
PV	0.988	0.971–1.003	0.121	0.989	0.973–1.005	0.171	1.033	1.018–1.048	**<0.01** *	1.029	1.014–1.044	**<0.01** *	1.017	1.004–1.031	**0.014** *	1.017	1.002–1.032	**0.025** *
PSA density	0.857	0.341–1.859	0.711				0.727	0.240–1.749	0.507				0.415	0.130–1.076	0.073	0.643	0.196–1.666	0.393
BMI	0.976	0.900–1.054	0.547				1.056	0.972–1.145	0.196				1.082	1.003–1.169	**0.040** *	1.062	0.983–1.148	0.128
Pre-op α1 blocker	1.102	0.473–2.363	0.812				1.863	0.819–3.967	0.132	1.628	0.710–3.735	0.263	1.102	0.473–2.363	0.812			
HT	1.253	0.804–1.949	0.317				1.401	0.863–2.274	0.172				1.021	0.653–1.591	0.924			
DM	0.856	0.446–1.560	0.620				1.266	0.654–2.336	0.472				1.751	0.987–3.044	0.055	1.658	0.910–2.962	0.097
Console time	1.001	0.995–1.004	0.763				1.005	1.001–1.010	**0.025** *	1.002	0.997–1.008	0.433	0.999	0995–1.003	0.627			
Blood loss	1.000	0.999–1.001	0.489				1.001	1.000–1.001	**0.025** *	1.001	0.999–1.001	0.220	0.999	0.999–1.001	0.827			
Nerve sparing	0.916	0.558–1.476	0.721				0.779	0.451–1.346	0.365				1.305	0.811–2.079	0.269			

*: statistically significant

Abbreviations pre-op PSA: preoperative prostate specific antigen, PV: preoperative prostate volume BMI: body mass index, HT: hypertension

DM: diabetes mellitus, ΔMFR: perioperative change in maximum flow rate, ΔVV: perioperative change in voided volume

ΔPVR: perioperative change in post-void residual urine, OR: odds ratio, CI: confidence interval

**Fig 4:**
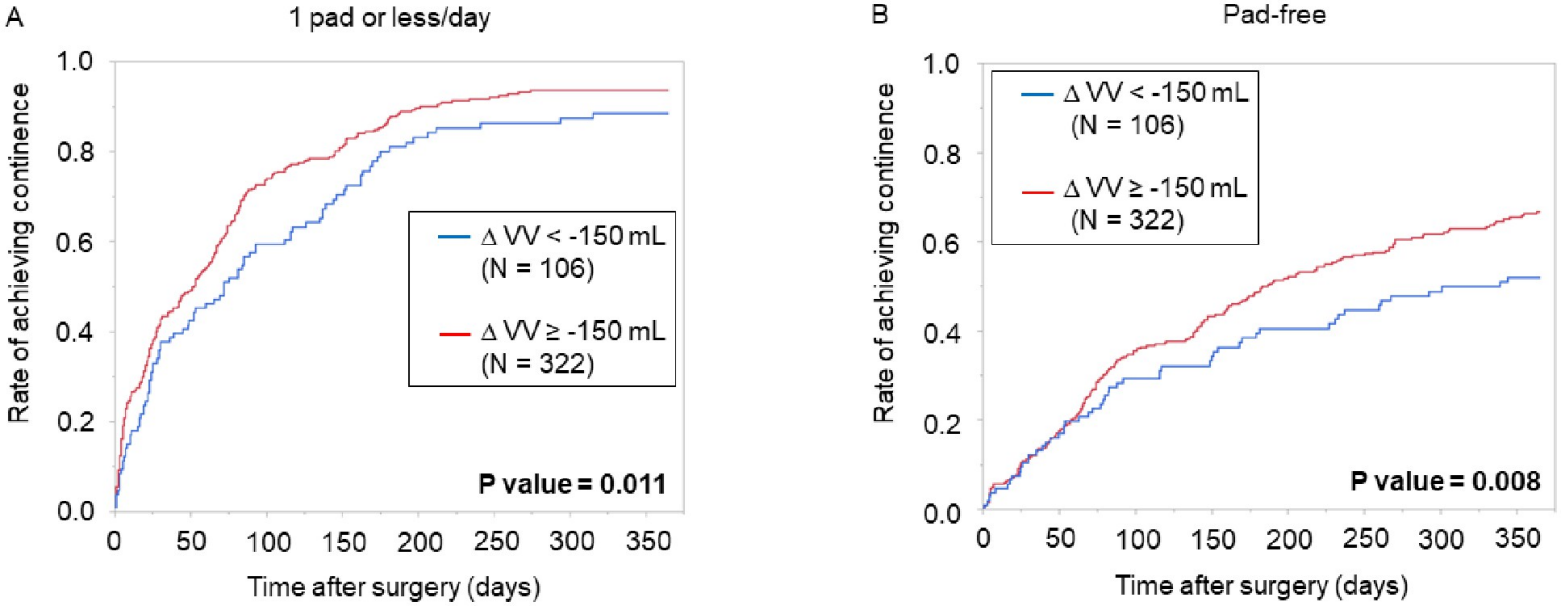
Post-RARP urinary continence recovery stratified by voided volume change. Kaplan-Meier curves for post-operative continence recovery to (A) use of 1 pad or less per day, and (B) pad-free status, as stratified by change in voided volume. A statistically significant difference was found for both curves, with a larger decrease in voided volume exhibiting a lower continence rate (log-rank test: P value = 0.011 and 0.008, respectively). Abbreviations RARP: robot-assisted radical prostatectomyΔVV: perioperative change in voided volume.

The perioperative change of the individual parameters of CLSS were analyzed for their association with UFM parameters (Table 4). A decrease in VV of over 150 mL led to increased urge and stress incontinence. A decrease in PVR of over 50 mL led to decreased daytime frequency, slow stream, and bladder pain. An increase in MFR of over 10 mL /s led to decreased slow stream and straining. All three UFM parameters led to a significant change in total CLSS score. Continence recovery curves were plotted, for recovery to either pad-free status, or one pad per day or less (Fig 4). When stratified by a decrease in VV of over/under 150 mL, a significant difference was seen in both curves, with a greater decrease in VV showing association with a delay in continence recovery.

**Table 4:** Perioperative changes in CLSS questionnaire results stratified by uroflowmetry parameter changes.

	ΔVV <-150mL	ΔVV ≥-150mL	P value	ΔPVR <-50mL	ΔPVR ≥-50mL	P value	ΔMFR >+10mL/s	ΔMFR ≤+10mL/s	P value
Daytime frequency	0.267	0.087	0.124	-0.086	0.182	**0.031** *	0.094	0.143	0.733
Nocturia	0.295	0.264	0.516	0.148	0.301	0.268	0.321	0.255	0.561
Urgency	0.057	0.012	0.755	-0.111	0.055	0.121	0.075	0.006	0.567
Urgency incontinence	0.743	0.413	**0.002** *	0.420	0.512	0.564	0.396	0.526	0.153
Stress incontinence	1.390	1.106	**0.014** *	1.086	1.197	0.288	1.236	1.155	0.486
Slow stream	-0.362	-0.385	0.735	-0.741	-0.295	**0.007** *	-0.877	-0.215	**<0.001** *
Straining	0.229	0.252	0.985	0.074	0.286	0.187	0.000	0.327	**0.005** *
Incomplete emptying	0.019	-0.137	0.221	-0.247	-0.064	0.156	-0.217	-0.059	0.174
Bladder pain	-0.067	0.047	0.066	-0.086	0.043	**0.037** *	0.028	0.016	0.521
Urethral pain	0.067	0.056	0.904	0.111	0.046	0.242	0.047	0.062	0.325
Total CLSS	2.613	1.683	**0.015** *	0.561	2.234	**0.009** *	1.000	2.214	**0.003** *

*: statistically significant

Values represent mean (post-RARP score–pre-RARP score)

Positive value = increased symptoms, negative value = decreased symptoms

Abbreviations CLSS: Core Lower urinary tract Symptom Score, ΔVV = perioperative change in voided volume

ΔPVR: perioperative change in post-void residual urine, ΔMFR: perioperative change in maximum flow rate

## Discussion

Radical prostatectomy drastically alters the anatomy and function of the bladder and urethra, and in turn leads to a variety of urological complications including urinary incontinence, voiding dysfunction, and storage dysfunction [4]. Although multiple reports state that the introduction of RARP has led to a general improvement of post-operative symptoms, post-operative LUTS remains an issue of importance [11, 12]. We ourselves have conducted several studies to evaluate pre- and post-operative LUTS, especially concerning post-prostatectomy incontinence (PPI) [13, 14]. We have reported on the longitudinal change of CLSS and other questionnaires post-RARP and found a short-term worsening of symptoms such as nocturia, urgency urinary incontinence, and stress urinary incontinence [15]. Furthermore, studies utilizing urodynamics have provided objective data giving us insight on the complex nature of the effect of prostatectomy. Factors such as direct surgical injury, bladder/sphincter denervation, or anastomotic stricture lead to varying degrees of sphincter deficiency/instability, detrusor overactivity/underactivity/instability, impaired bladder compliance, and bladder outlet obstruction (BOO), which in turn lead to the actual symptoms such as PPI [3–7, 16–21]. The urodynamic findings attributed to these changes, such as decreased maximum urethral closing pressure, decreased functional profile length, and decreased maximum detrusor pressure at MFR have all been linked to the prevalence of PPI or a delay in its recovery [3–7, 16–21]. However, urodynamic studies remain an invasive and time-consuming method, and further exploration on less invasive testing such as UFM is warranted.

Our results showed a significant increase of MFR and decrease of VV and PVR. This is in line with past reports on post-prostatectomy UFM results [22–25]. The changes in MFR and PVR particularly seem to reflect the alleviation of BOO induced by prostate resection. This is reinforced by reports in which urodynamic studies revealed a decline of the BOO index after surgery [26]. The extent to which changes in uroflowmetry affect urinary bother may be up for debate, but our cohort exhibited an association with the changes in MFR and PVR to clinical symptoms. The sole symptom of CLSS that showed improvement post-RARP was slow stream, and this study showed that both MFR and PVR were associated with the improvement of the slow stream component. It stands to reason that PV was found to be an independent predictor for both MFR increase and PVR decline, as a larger prostate would usually lead to more severe BOO, and its resection would lead to a larger improvement in voiding parameters. The cutoff value of PV was approximately 30 mL and may be used to inform patients on post-operative improvement of slow stream. Interestingly, age was also found to be a negative predictor of an improvement in MFR of over 10 mL/s. Some studies have reported on a decrease in detrusor contractility with age, which may be a reason for MFR showing less improvement after RARP in older men [27].

The decrease of VV, on the other hand, was associated with both CLSS incontinence components, and also a delay in recovery from incontinence. It is natural to postulate that VV decreases after RARP, since the reduction of bladder capacity to a varying degree is inevitable when bladder neck is dissected in such a procedure. Were this to be the case, it is expected that PV would be a predictor of the decrease of VV, in that a larger prostate would likely lead to a larger resection of the bladder neck. Interestingly, we could not identify any pre-operative factors including PV that contributed to this decrease in VV from our cohort. No previous studies have conducted multivariate analysis to identify predictors of the changes in UFM parameters, and just one report found a relation between BMI and decrease in VV [28]. In past reports, post-RARP LUTS have been attributed to such factors as sphincteric dysfunction/instability, detrusor overactivity/underactivity, and impaired compliance [3–7, 13, 16–21]. Post-operative detrusor function varies greatly depending on the case and may be either over- or under-active, the latter possibly leading to an increase in VV rather than a decrease. However, most studies seem to agree that the effects of sphincteric dysfunction/instability outweigh bladder dysfunction in the majority of cases. Therefore, it stands to reason that sphincteric dysfunction/instability would lead to an impairment of bladder storage function, and a consequent decrease in VV, even in the presence of detrusor underactivity. As noted before, PPI has also been attributed to sphincteric dysfunction/instability, and this is likely the reason why an association between VV decrease and PPI prevalence was observed in our cohort. Taken together with the improvement of BOO, it is of note that the short-term CLSS and QOL indices were both worsened from pre-surgery, which indicate that the negative effect of PPI seems to have affected patients more strongly than the positive effect of the amelioration of BOO. This is easily understandable in that PPI constitutes a large percentage of complaints in post-RARP patients, as previously reported in many reports including our own [15]. Previous reports of LUTS improving after RARP presumably represent a reversal of this relationship, with the recovery of PPI leading to amelioration of BOO becoming more prominent [11].

Several recent studies have investigated intra-operative procedures affecting post-operative morbidity, perhaps due to the flexibility of surgical procedures afforded by the introduction of RARP. Techniques such as nerve sparing, bladder neck preservation, puboprostatic ligament preservation, rhabdosphincter reconstruction, bladder neck plication, and retropubic suspension have been reported to lead to improvement in PPI [29–34]. Haga et al. found that nerve-sparing led to an increased maximum VV and improvement of post-operative LUTS [30]. Although nerve sparing did not directly correlate with the amount of VV decrease in our cohort, it may be beneficial to examine further the effect of surgical techniques such as bladder neck preservation or bladder neck plication on VV. The preservation of a funneled bladder neck attained by these two techniques is said to lead to less stretch on the bladder neck and consequently to preservation of bladder function [29, 31, 33].

Some limitations of the present study should be mentioned. First of all, the retrospective nature of the study design at a single institution may have biased the results. Especially considering the aforementioned complexity of post-prostatectomy changes, a different or larger cohort may have led to different results or cutoff values, similarly to how previous urodynamics studies have led to differing predictors of PPI. Further accumulation of evidence is crucial in confirming our results. Secondly, UFM has traditionally been considered to be reliable when the VV is above 150mL. In two Japanese studies of post-RARP UFM conducted by a single group, median VV was approximately 250mL preoperatively and 150~170mL postoperatively, similar to our own result [22, 35]. Based on these reports we decided not to exclude patients with VV under 150mL, but decided to use perioperative change in UFM parameters, thereby ameliorating this possible bias caused by ethnicity. We also considered the use of VV beneficial to our study in that it reflected natural micturition, because the endpoints of CLSS and incontinence are both reflective of natural micturition as opposed to a controlled storage until maximum urge as in urodynamics. Perhaps due to this difference, there have been previous reports that UFM results with VV below 150mL also correlated well with storage symptoms such as number of voids, nocturnal voids, and maximum VV [36].

## Conclusion

We examined the results of routine UFM conducted before and after prostatectomy, and found a significant increase in MFR and decrease in VV and PVR. PV was an independent predictor of the changes in MFR and PVR. These changes significantly affected improvement of voiding symptoms such as slow stream and straining, and were considered to be representative of the amelioration of BOO upon prostatectomy. The decrease in VV was associated with a worsening of stress/urge incontinence symptoms, and also a delay in incontinence recovery.

### Author contributions

YY designed the study and supervised the process of completing the study. YT and YY performed statistical analysis and drafted the manuscript. KT, NK, YH, JM, and YY contributed to data acquisition. ST, YA, YS, TK, DY, and TF assisted in data interpretation and critical revision of the manuscript. HK assisted in data interpretation, critical revision of the manuscript, and administrative supervision. All authors read and approved the final manuscript.

## Supporting information

S1 Data (XLSX)

S1 File (DOCX)

S1 Table Patient demographics stratified by perioperative change in voided volume (ΔVV). (DOCX)

S2 Table Patient demographics stratified by perioperative change in post-void residual urine (ΔPVR). (DOCX)

S3 Table Patient demographics stratified by perioperative change in maximum flow rate (ΔMFR). (DOCX)
