# Supplementary material for: The association between the parameters of uroflowmetry and lower urinary tract symptoms in prostate cancer patients after robot-assisted radical prostatectomy
Source: PLoS One. 2022 Oct 6;17(10):e0275069. doi: 10.1371/journal.pone.0275069 (PMC9536545; doi:10.1371/journal.pone.0275069)
Supplement: S1 File — (DOCX) [file pone.0275069.s002.docx]

**Table for Reviewers** List of all PubMed articles for “prostatectomy” and “uroflowmetry”

| No | Author | Inst | Year | No of RARP cases | modality | test methods | timing of tests (months post-op) | Summary |
| --- | --- | --- | --- | --- | --- | --- | --- | --- |
| 1 | Majima | Nagoya | 2021 | 313 | RARP | urethral pressure profile | pre- | low pre-MUCP→age・PV・ED→continence recovery |
| 2 | Walker | Sydney | 2021 | 85 | RARP/RP | UDS | pre- | OAB / filling phase abnormality correlation |
| 3 | Iguchi | Osaka | 2020 | 75 | RARP | UDS | pre-, post-(3) | low pre-MUCP・pre- and post-DO→incontinence |
| 4 | Huang | Taipei | 2019 | 48 | LRP | UDS | pre-, post-(1,3) | (bladder neck), functional profile length → incontinence |
| 5 | Matsukawa | Nagoya | 2018 | 245 | RARP | IPSS, OABSS, UDS(UPP) | pre-, post-(3) | low pre-IPSS-QOL, low post MUCP → de novo OAB |
| 6 | Ruiz |  | 2018 | review |  |  |  |  |
| 7 | Kitta | Hokkaido | 2017 | 37 | RP/LRP | PFS, IPSS, QOL | pre-, post-(12) | Qmax↑, PdetQmax↓, RV↓, QOL improved |
| 8 | Kadono | Kanazawa | 2016 | 84 | RARP | UDS, MRI | pre-, post-(1,12) | (nerve sparing)→MUCP, FPL: no effect on bladder function |
| 9 | Alenizi | Montreal | 2015 | 108 | RARP | UFM(+stop test) | post(1wk) | positive UFM stop test →　early recovery of continence |
| 10 | Kadono | Kanazawa | 2015 | 63 | RARP | UDS | pre-, post-(1,12) | void↑　storage↓↑　sphincter↓ |
| 11 | Holm | Norway | 2014 | 94 | RP | UDS | post (years after) | UDS results→no correlation with PPI op(sling, artificial sphincter) |
| 12 | Yanagiuchi | Kobe | 2014 | 84 | RARP | UDS | pre- | DO, low MUCP → early incontinence |
| 13 | Kadono | Kanazawa | 2013 | 87 | RARP | UDS | pre-, post-(1) | low max capacity, low MUCP, low ALPP → incontinence |
| 14 | Cameron | Michigan | 2015 | 26 | RP/RARP | UDS, MRI | post (>1 year) | short urethra、sphincter distortion→PPI |
| 15 | Radomski | Toronto | 2013 | review |  |  |  | UDS：limited access, not predictive |
| 16 | Barnoiu | Spain | 2014 | 58 | RARP | UDS | pre- | low bladder compliance, low MUCP, BOO →PPI |
| 17 | dos Reis | Brazil | 2013 | 88 | RP | UDS | post (>1 year) | questionnaire:no correlation,　sphincter disfunction→PPI |
| **18** | **Mitsui** | **Hokkaido** | **2012** | **43** | **RP/LRP** | **UFM, PFS** | **UFM(pre,3,6,12)** | **MFR/PVR improved, capacity/contractility/IPSS unchanged** |
| 19 | Dubbelman | Netherlands | 2012 | 66 | RP | UFM | pre-, post-(6) | DO→PPI　univariate analysis only |
| 20 | Chung | MSKCC | 2013 | 264 | RP | UDS | post- | DU in 41%, BOO in 17%, DO in 27% |
| 21 | Bosch | Utrecht | 2011 | review |  |  |  | UDS relevance in question. PPI prediction fair |
| 22 | Matsukawa | Nagoya | 2010 | 110 | LRP | UDS | pre-, post-(3-5) | MUCP, Pdet max decrease, max cap/storage unchanged |
| 23 | Song | Seoul | 2010 | 72 | RP | UDS | pre-, post-(3,6,36) | Max capacity, MUCP, Pdet max decrease, de novo DO |
| 24 | Matsukawa | Nagoya | 2009 | 63 vs58 | RP vs LRP | UDS | pre-, post-(3-9) | low MUCP, short FPL, low compliance, DO→PPI |
| 25 | Giannantoni | Italy | 2007 | 54(32) | RP | UDS | pre-, post-(8, 36) | bladder compliance↓,hypodetrusor↑, sphincterdysfunction |
| 26 | Porena | Italy | 2007 | review | RP |  |  | DO, compliance↓, no analysis on BOO |
| 27 | Namiki | Tohoku | 2006 | 225 | RP | IPSS only | pre-,post-(3,6,12,18,24) | symptoms improve, function decline |
| 28 | Majoros | Hungary | 2006 | 63 | RP | UDS | pre-, post-(8) | high MUCP continent; PPI from sphincter disfunc |
| 29 | Keilb | Chicago | 2005 | 146 | RP | VUDS | post (>1 year) | sphincter>compliance/detrusor as reason for PPI |
| 30 | Natsume | Nara | 2004 | 17 | RP | PFS | pre-, post-(1,3,6) | de novo detrusor underactivity →　incontinence |
| 31 | Giannantoni | Italy | 2004 | 49 | RP | UDS | pre-, post-(1,8) | bladder compliance↓,detrusor hypo↑, DO-sphincter disfunction correlation. |
| **32** | **Kumar** | **UK** | **2003** | **50** | **RP** | **UFM, IPSS** | **pre-, post-(3,6)** | **MFR/PVR improves in mod-severe LUTS patients** |
| **33** | **Masters** | **NZ** | **2003** | **125** | **RP** | **UFM, IPSS** | **pre-, post-(2,6,14,20)** | **MFR↑, IPSS improvement** |
| 34 | Aherling |  | 2003 | 100 | RP | UFM、IPSS | post (1-9years) | MFR better than historical control |
| 35 | Groutz | New York | 2000 | 83 | RP | VUDS | post (1-10years) | sphincter disfunc8%, detrusor34% |
| 36 | John | Switz | 2000 | 34 | RP | UDS | pre-, post-(1,6) | urethral sensitivity, pressure transmission →　PPI |
| 37 | Zermann | Germany | 2000 | 18 | RP | UDS, EMG, neurological | pre-, post-(0.5,6) | study on pelvic floor muscle voltage |
| 38 | Kleinhans | Germany | 1999 | 66(44) | RP | UDS | pre-, post-(avg7.6) | no apparent predictors of PPI, post-op MUCP↓ |
| 39 | Winters | Louisiana | 1998 | 65(+27TURP) | RP | VUDS | post- | VLPP92%, detrusor37%, unrelated to PPI |
| 40 | Hammerer | Germany | 1997 | 82 | RP | UDS | pre-, post-(1,6) | func.length↓MUCP↓→PPI　improv at 6m 　max.cap.↓ |
| 41 | Minervini | Italy | 1996 | 39 | RP | UDS | pre-, post-(1,6) | func.length↓MUCP↓→PPI |
| 42 | Aboseif | California | 1994 | 92 | RP | UDS | pre- | detrusor+sphincter instability →PPI |
| 43 | Kurimura | Fukushima | 2020 | 329 | RARP | pad T, UFM, quest, US | pre-, post-(1,3,6,9,12) | preop-incontinence→VV↓,PVR↓, MFR unchanged |
| 44 | Juszczak | Poland | 2019 | 70 | RP | UFM, US, IPSS, OABSS | pre- | pre-OAB→PPI |
| 45 | Saldi | Italy | 2018 | 112 | RP+RT | UFM | pre-, post-(6) | salvage RT→decreased MFR |
| 46 | Ervandian | Denmark | 2018 | 16 | RP+sRT | UFM, PFS, UPP (=UDS) | post- (6-10 years) | SRT→compliance, max capacity, BOO |
| 47 | Haga | Fukushima | 2016 | 200 | RARP | UFM, padT, FVC,ques | pre-, post-(3,12) | storage↓, VV↓, pad↑, PVR↓, IIEF↓ |
| 48 | Haga | Fukushima | 2016 | 100 | RARP | UFM, padT,FVC,ques | pre-, post-(3,12) | Vesical adaptation response to diuresis |
| 49 | Yang | Daejeon | 2016 | 50 | RP | UFM, IPSS, QOL | pre-, post-(12) | storage symptoms worsen anticholin effective |
| 50 | Yabe | Fukushima | 2016 | 80 | RARP | UFM, IPSS, padT, FVC | pre-, post-(1,3,6) | arterial sclerosis→LUTS recovery delay |
| 51 | Haga | Fukushima | 2014 | 50 | RP/LRP | UFM, MRI, IPSS, continence | pre-, post-(6+) | func.length・UVJ position・urinary pooling →　PPI |
| 52 | El-Hakim | Montreal | 2014 | 108 | RARP | UFM(+stop test) | post(at cath removal） | stop test→early recovery of continence |
| 53 | Jeong | Seoul | 2014 | 236 | RARP | IPSS, UFM | pre-, post-(2wk) | tamsulosin →　retention rate↓ |
| 54 | Mucciardi | Italy | 2013 | 100 | RP | UDS, UFM | pre-, post-(UDS12) (UFM3,6) | detrusor underactivity →　bladder neck contraction |
| 55 | Skarecky | California | 2011 | 172 | RARP | UFM | pre- | no preop parameters predict PPI, only pad status after catheter removal |
| 56 | Ishida | Nagoya | 2008 | 80 | LRP | UDS | pre-, post-(2-6) | PdetQmax↓, Qmax↑ |
| **57** | **Ahlering** | **California** | **2004** | **100** | **RARP** | **AUASS, UFM, PVR, IIEF** | **pre-, post- (3)** | **high BMI → longer op. time, blood loss↑, PPI↑, LUTS, VV↓** |
| 58 | Kanno | Toyooka | 2004 | 35 | RP | UFM | post (at cath removal、plus 4-20） | 34％BOO　→　recommended check with UFM |
| 59 | Igarashi | Tokyo | 2004 | 43 | RP | UFM, cystometry, IPSS | pre-, post-(1,3,6,12) | improvement after 3-6m; MFR↑, PVR↓, VV unchanged |
| 60 | Goluboff | New York | 1996 | 25 | RP | UFM, PFS | post | detrusor instability prevalent |
| 61 | Ando | Tokyo | 1994 | 18 | RP | UDS | pre-, post-(1,3,6,12) | FPL, MUCP↓, low compliance at 1m, recovery at 12m |

Articles indicated in red or blue (13 articles): specifically conducted UFM both before and after prostatectomy.

Articles indicated in red (7 articles): reported a change in any of VV, MFR, or PVR.

Articles indicated in bold red (4 articles): analyzed a relation between the change in UFM parameters and other clinical parameters
