# Supplementary material for: The association between the parameters of uroflowmetry and lower urinary tract symptoms in prostate cancer patients after robot-assisted radical prostatectomy
Source: PLoS One. 2022 Oct 6;17(10):e0275069. doi: 10.1371/journal.pone.0275069 (PMC9536545; doi:10.1371/journal.pone.0275069)
Supplement: S1 Table — (DOCX) [file pone.0275069.s003.docx]

**S1 Table. Patient demographics stratified by perioperative change in voided volume (ΔVV)**

| Parameters (N=428) | | ΔVV < 150mL (N=106) | ΔVV ≥ 150mL(N=322) | P value |
| --- | --- | --- | --- | --- |
| Age (years) |  | 66(62-71) | 68(64-71) | 0.106 |
| Pre-operative PSA (ng/mL) |  | 7.0(5.4-9.8) | 7.7(5.6-11.0) | 0.144 |
| Prostate volume (mL) |  | 26.5(20.8-36.0) | 27.0(21.9-37.0) | 0.174 |
| PSA density (ng/mL^2^) |  | 0.28(0.19-0.40) | 0.28(0.19-0.40) | 0.883 |
| BMI (kg/m^2^) |  | 23.4(21.9-25.4) | 23.7(22.0-25.3) | 0.635 |
| D'Amico risk classification | Low | 24(22.6%) | 40(12.4%) | **0.011*** |
|  | Intermediate-high | 82(77.4%) | 282(87.6%) |  |
| Pre-operative α1 blocker | No | 97(91.5%) | 297(92.2%) | 0.810 |
|  | Yes | 9(8.5%) | 25(7.8%) |  |
| HT | absent | 57(53.8%) | 191(59.3%) | 0.316 |
|  | present | 49(46.2%) | 131(40.7%) |  |
| DM | absent | 91(85.9%) | 270(83.9%) | 0.623 |
|  | present | 15(14.1%) | 52(16.2%) |  |
| Console time (min) |  | 170(130-208) | 169(130-202) | 0.805 |
| Blood loss (mL) |  | 300(150-500) | 250(100-500) | 0.416 |
| Nerve sparing | none | 76(71.7%) | 225(69.9%) | 0.431 |
|  | unilateral | 30(28.3%) | 92(28.6%) |  |
|  | bilateral | 0(0%) | 5(1.6%) |  |
| pT stage | T2 | 73(68.9%) | 224(74.8%) | 0.893 |
|  | T3 | 33(31.1%) | 98(25.2%) |  |
| CLSS | total | 5(3-8) | 5(3-8) | 0.825 |
| QOL index |  | 3(1-4) | 3(2-4) | 0.389 |
| * : statistically significant | |  |  |  |
| median value(IQR) or number of cases(%) | | |  |  |
| Abbreviations ΔVV : preoperative voided volume – postoperative voided volume | | | | |
| PSA: prostate-specific antigen, BMI: body mass index, HT: hypertension, DM: diabetes mellitus | | | | |
| pT stage: pathological T stage, CLSS: core lower urinary tract symptom score, QOL index: quality of life index | | | | |
