# Supplementary material for: The association between the parameters of uroflowmetry and lower urinary tract symptoms in prostate cancer patients after robot-assisted radical prostatectomy
Source: PLoS One. 2022 Oct 6;17(10):e0275069. doi: 10.1371/journal.pone.0275069 (PMC9536545; doi:10.1371/journal.pone.0275069)
Supplement: S2 Table — (DOCX) [file pone.0275069.s004.docx]

**S2 Table. Patient demographics stratified by perioperative change in post-void residual urine (ΔPVR)**

| Parameters (N=428) | | ΔPVR < -50mL (N=82) | ΔPVR ≥ -50mL (N=346) | P value |
| --- | --- | --- | --- | --- |
| Age (years) |  | 69(64-71) | 67.5(63-71) | 0.332 |
| Pre-operative PSA (ng/mL) |  | 8.1(5.5-11.6) | 7.4(5.6-10.6) | 0.254 |
| Prostate volume (mL) |  | 33(22-47.3) | 27.0(21-35) | **<0.001*** |
| PSA density (ng/mL^2^) |  | 0.24(0.17-0.39) | 0.29(0.20-0.40) | 0.142 |
| BMI (kg/m^2^) |  | 24.2(22.4-26.1) | 23.7(21.9-25.2) | 0.111 |
| D'Amico risk classification | Low | 13(15.9%) | 51(14.7%) | 0.800 |
|  | Intermediate-high | 69(84.2%) | 295(85.3%) |  |
| Pre-operative α1 blocker | No | 72(87.8%) | 322(93.1%) | 0.113 |
|  | Yes | 10(12.2%) | 24(6.9%) |  |
| HT | absent | 42(51.2%) | 206(59.5%) | 0.170 |
|  | present | 40(48.8%) | 140(40.5%) |  |
| DM | absent | 67(81.7%) | 294(85.0%) | 0.465 |
|  | present | 15(18.3%) | 52(15.0%) |  |
| Console time (min) |  | 173(152-208) | 163(128-204) | **0.035*** |
| Blood loss (mL) |  | 300(105-600) | 250(100-500) | 0.115 |
| Nerve sparing | none | 61(74.4%) | 240(69.4%) | 0.656 |
|  | unilateral | 20(24.4%) | 102(29.5%) |  |
|  | bilateral | 1(1.2%) | 4(1.2%) |  |
| pT stage | T2 | 60(73.2%) | 237(68.5%) | 0.409 |
|  | T3 | 22(26.8%) | 109(31.5%) |  |
| CLSS | total | 7(4-9) | 5(3-8) | **0.004*** |
| QOL index |  | 3(2-5) | 3(1-4) | **0.004*** |
| * : statistically significant | |  |  |  |
| median value(IQR) or number of cases(%) | | |  |  |
| Abbreviations ΔPVR : preoperative post-void residual urine – postoperative post-void residual urine | | | | |
| PSA: prostate-specific antigen, BMI: body mass index, HT: hypertension, DM: diabetes mellitus | | | | |
| pT stage: pathological T stage, CLSS: core lower urinary tract symptom score, QOL index: quality of life index | | | | |
