# Supplementary material for: The association between the parameters of uroflowmetry and lower urinary tract symptoms in prostate cancer patients after robot-assisted radical prostatectomy
Source: PLoS One. 2022 Oct 6;17(10):e0275069. doi: 10.1371/journal.pone.0275069 (PMC9536545; doi:10.1371/journal.pone.0275069)
Supplement: S3 Table — (DOCX) [file pone.0275069.s005.docx]

**S3 Table. Patient demographics stratified by perioperative change in maximum flow rate (ΔMFR)**

| Parameters (N=428) | | ΔMFR >10mL/s (N=106) | ΔMFR ≤ 10mL (N=322) | P value |
| --- | --- | --- | --- | --- |
| Age (years) |  | 66(61.8-70) | 68(64-71) | **0.039*** |
| Pre-operative PSA (ng/mL) |  | 7.3(5.6-10.4) | 7.6(5.5-11.0) | 0.638 |
| Prostate volume (mL) |  | 28(22-41.25) | 27(21-36) | 0.109 |
| PSA density (ng/mL^2^) |  | 0.26(0.18-0.35) | 0.29(0.20-0.42) | 0.096 |
| BMI (kg/m^2^) |  | 24.2(22.7-26.0) | 23.6(21.8-25.3) | **0.023*** |
| D'Amico risk classification | Low | 17(16.0%) | 47(14.6%) | 0.718 |
|  | Intermediate-high | 89(84.0%) | 275(85.4%) |  |
| Pre-operative α1 blocker | No | 97(91.5%) | 297(92.2%) | 0.810 |
|  | Yes | 9(8.5%) | 25(7.8%) |  |
| HT | absent | 61(57.6%) | 187(58.1%) | 0.924 |
|  | present | 45(42.5%) | 135(41.9%) |  |
| DM | absent | 83(78.3%) | 278(86.3%) | **0.048*** |
|  | present | 23(21.7%) | 44(13.7%) |  |
| Console time (min) |  | 162(128-203) | 170(135-206) | 0.596 |
| Blood loss (mL) |  | 275(100-500) | 250(100-500) | 0.891 |
| Nerve sparing | none | 70(66.0%) | 231(71.7%) | **0.013*** |
|  | unilateral | 32(30.2%) | 90(28.0%) |  |
|  | bilateral | 4(3.8%) | 1(0.3%) |  |
| pT stage | T2 | 84(79.3%) | 213(66.2%) | **0.011*** |
|  | T3 | 22(20.8%) | 109(33.9%) |  |
| CLSS | total | 5(4-8) | 5(3-8) | 0.487 |
| QOL index |  | 3(1-4) | 3(2-4) | 0.262 |
| * : statistically significant | |  |  |  |
| median value(IQR) or number of cases(%) | | |  |  |
| Abbreviations ΔMFR : preoperative maximum flow rate – postoperative maximum flow rate | | | | |
| PSA: prostate-specific antigen, BMI: body mass index, HT: hypertension, DM: diabetes mellitus | | | | |
| pT stage: pathological T stage, CLSS: core lower urinary tract symptom score, QOL index: quality of life index | | | | |
